# Pharmacokinetics of Piperacillin in an Experimental Porcine Liver Model During Normothermic Machine Perfusion

**DOI:** 10.3389/ti.2025.15348

**Published:** 2026-01-06

**Authors:** Simon Mathis, Gabriel Putzer, Judith Martini, Thomas Resch, Christina Bogensperger, Michael Dullnig, Jonas Dunz, Fariha Nawabi, Nikolai Staier, Magdalena Bordt, Theresa Hautz, Julia Hofmann, Stefan Schneeberger, Christoph Dorn

**Affiliations:** 1 Department of Anaesthesiology and Critical Care Medicine, Medical University of Innsbruck, Innsbruck, Austria; 2 Department of Visceral, Transplant and Thoracic Surgery, OrganLife™ Organ Regeneration Center of Excellence, Medical University of Innsbruck, Innsbruck, Austria; 3 Institute of Pharmacy, University of Regensburg, Regensburg, Germany

**Keywords:** normothermic machine perfusion, piperacillin, microdialysis, liver transplant, pharmacokinetic analysis

Dear Editors,

Normothermic machine perfusion (NMP) has become a routine technique in liver transplantation, allowing preservation and assessment of grafts prior to implantation [1]. Current commercially approved systems are limited to 24 h, prompting interest in prolonged perfusion to further improve graft conditioning. During extended NMP, microbial contamination is a potential risk, as warm, humid conditions promote bacterial growth [2]. To mitigate this, antimicrobials are commonly added to the perfusate, although the pharmacokinetics under NMP conditions - characterized by a small volume of distribution and absence of renal clearance - remain insufficiently understood.

Piperacillin, a broad-spectrum β-lactam, has been used experimentally for extended NMP [3]. The present study characterizes its pharmacokinetics during liver NMP, including perfusate levels, tissue concentrations via microdialysis, and bile excretion.

In this study, the livers of eight domestic pigs were used and perfused with 1,500 mL leukocyte-depleted whole blood from the donor animal. A microdialysis double lumen catheter with a semi-permeable membrane at the tip was inserted into the liver tissue. Prior to piperacillin administration, the relative recovery of each microdialysis probe was determined by retrodialysis using Ringer’s solution (Fresenius Kabi Austria GmbH, Graz, Austria) containing 80 μg mL^−1^ piperacillin. The piperacillin concentrations in the retroperfusate (C_RP_) and the corresponding retrodialysate (C_RD_) were used to calculate the relative recovery using the formula: relative recovery = [1 - (C_RD_/C_RP_)] × 100%.

After calibration, 400 mg piperacillin was added into the reservoir of the NMP system.

The catheter was perfused with Ringer’s solution at a flow rate of 1 μL min^−1^ to facilitate the exchange of piperacillin between the liver interstitial space fluid (ISF) and the microdialysis perfusate across the membrane. The piperacillin concentration measured in the resulting microdialysate (C_MD_), corrected for the relative recovery of the probe, was used to estimate the piperacillin concentration in the ISF (C_ISF_ = C_MD_ × 100%/relative recovery). Microdialysate samples were collected at 20-minute intervals for 2 h and at 60-minute intervals for up to 8 h after piperacillin administration. NMP perfusate samples were collected 5 min, 15, 30, 60 min and 3, 4, 6 and 24 h after piperacillin administration. Bile samples were analyzed 4 and 8 h after piperacillin administration (in four grafts due to technical limitations). The concentrations of piperacillin were determined by HPLC-UV.

Stable perfusion and organ function was achieved over the entire study period. The piperacillin concentration in perfusate samples and ISF of liver tissue during NMP are shown in Figure 1. After piperacillin application (t = 0 min), the first samples of NMP perfusate, taken at 5 min (n = 5) or 15 min (n = 3) showed the highest measured concentrations of total piperacillin: 109.9 ± 25.1 mg L^−1^ at 5 min (n = 5) or 78.7 ± 29.9 mg L^−1^ at 15 min (n = 3; corresponding to 108.5 ± 36.7 mg L^−1^ at 5 min).

**FIGURE 1:**
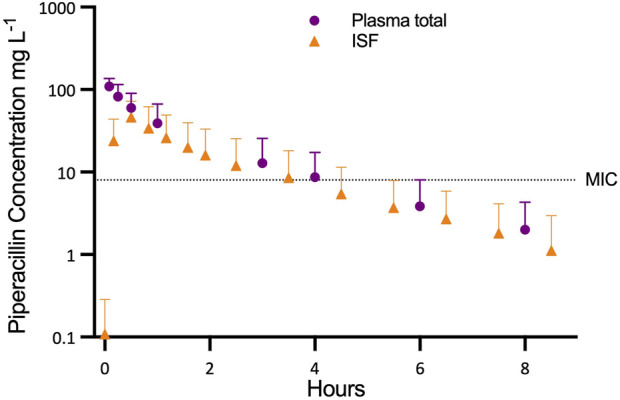
Progression of piperacillin concentration in plasma and interstitial fluid (ISF) in relation to the minimum inhibitory concentration (MIC).

The mean area under the curve (AUC(0.8)) for total piperacillin in NMP perfusate was 135 ± 92.91 mg L^−1^ h, AUC_INFINITY_ was 144 ± 103 mg L^−1^ h, the elimination half-life time was 1.43 ± 0.42 h. The mean volume of distribution was 8.22 ± 4.83 L and the mean clearance of piperacillin was 4.54 ± 3.25 L h^−1.^

The unbound fraction (fu) of piperacillin in NMP perfusate was 93.8% ± 1.31%. Mean *f*AUC(0.8) and *f*AUC_INFINITY_ was 128 ± 86.6 mg L^−1^ h and 136 ± 95.9 mg L^−1^ h, respectively.

Relative recovery of the microdialysis probes was high (95.4% ± 4.67%) and ranged from 83.0% to 100%. Peak concentrations (C_max_) in ISF were reached at 10 min (n = 1), 30 min (n = 5) or after 50 min (n = 2). Mean C_max_ in ISF amounted to 48.6 ± 26.4 mg L^−1^. Mean elimination half-life (1.50 ± 0.36 h) was similar to that of the NMP perfusate.

Mean AUC(0.8) and AUC_INFINITY_ for piperacillin in ISF (87.18 ± 76.18 and 90.8 ± 81.3 mg L^−1^ h) were lower than in NMP perfusate (p = 0.016). The penetration ratio, defined as the ratio of the AUC_INFINITY_ for piperacillin in ISF to AUC_INFINFINITY_ for free piperacillin in NMP perfusate (AUC_INFINITY_ISF_/*f*AUC_INFINITY_perfusate_), was 0.653 ± 0.300.

The grafts produced 118.25 (±44.74) mL of bile during the first 8 h of perfusion with a mean piperacillin concentration of 1.48 ± 1.11 g L^−1^, corresponding to 168.15 ± 68.4 mg of piperacillin excreted in bile per graft.

Piperacillin rapidly achieved high concentrations in graft tissue during NMP, followed by swift elimination. Perfusate levels consistently exceeded tissue concentrations, with elimination predominantly occurring via bile in the absence of renal excretion. The reduced volume of distribution inherent to isolated liver perfusion, combined with rapid recirculation of a small perfusate volume, explains both the fast tissue penetration and rapid clearance.

Although piperacillin elimination *in vivo* is largely renal, biliary excretion represents a known alternative route, particularly in renal insufficiency [4]. This pathway likely accounts for the significant biliary concentrations observed in this study despite absent renal clearance.

Protein binding in NMP perfusate was markedly lower than in human plasma (20%–30%), attributed to the leukocyte-depleted whole blood used here [5]. In clinical NMP with red cell concentrates and colloids [6], protein binding would be negligible.

Using the EUCAST minimum inhibitory concentration breakpoint for piperacillin/tazobactam-sensitive strains (8 mg L^−1^), graft tissue levels were below this threshold within 4 hours of receiving a 400 mg bolus dose [7]. This suggests that a single-dose strategy provides only transient antimicrobial protection during NMP. Continuous infusion could maintain therapeutic levels, but dosing must account for the high inter-graft variability observed (coefficient of variation 72%).

These findings underscore that drug pharmacokinetics during NMP differ markedly from *in vivo* conditions, necessitating dedicated dosing studies for medications administered in this setting. Limitations include the use of an animal model [8], perfusate composition differing from clinical practice (although protein binding was low even with whole blood), and the lack of metabolite measurements.

In summary, piperacillin during NMP demonstrates rapid hepatic penetration and biliary elimination, with therapeutic levels declining within 4 hours. For prolonged or long-term NMP, continuous dosing strategies may be required to ensure sustained antimicrobial protection.

## Data Availability

The raw data supporting the conclusions of this article will be made available by the authors, without undue reservation.
