# Supplementary material for: Pharmacokinetics of Piperacillin in an Experimental Porcine Liver Model During Normothermic Machine Perfusion
Source: Transpl Int. 2026 Jan 6;38:15348. doi: 10.3389/ti.2025.15348 (PMC12815883; doi:10.3389/ti.2025.15348)
Supplement: Supplementary file 1 [file Supplementaryfile1.docx]

***Supplementary Material***

*Animal Preparation*

Preparation, induction of anesthesia and anesthetic management have previously been described in detail.[1] According to local laboratory standard, a preoperative dose of 1 g of meropenem and 1 g of vancomycin was administered to the animal. The femoral arteries of the pig were punctured bilaterally under ultrasound guidance, and a catheter (High Flow Dreilumen Katheter Set, Achim Schulz-Lauterbach GmbH, Vienna, Austria) and a leader cut (Microseld PTFE 4F 11cm, intra special catheters, Rehlingen-Siersburg, Germany) were inserted using the Seldinger technique. An arterial blood pressure measurement was obtained via the leader cut and norepinephrine was administered to ensure a mean arterial pressure (MAP) > 60 mmHg during the organ harvesting process. A total of 1,500 mL of whole blood was collected from the animal via the femoral catheter into sterile citrate bags. The intravascular volume deficit was then replaced with Elomel (Fresenius Kabi GmbH, Graz, Austria) and Gelofusin (Braun Melsungen AG, Melsungen, Germany) IV.

*Organ retrieval and NMP*

Following the systemic administration of 500 IU kg^-1^ of heparin. The warm ischemia period, with a MAP < 50 mmHg, lasted a maximum of four minutes before perfusion with 5,000 mL of Custodiol® (Dr. Franz Köhler Chemie, Bensheim, Germany). Subsequent to the retrieval of the liver, the vena cava, the hepatic artery, the portal vein and the bile duct of the graft were cannulated. Simultaneously, NMP was commenced by use of the OrganOx metra device (metra®, OrganOx, Oxford, UK). Technically, with the exception of cannulating the upper vena cava instead of the lower vena cava, this was performed in analogy to clinically used center standards, which have been described elsewhere. [2-4] In order to exclude the possibility of blood group incompatibility, the NMP perfusate consisted of 1,500 mL leukocyte-depleted whole blood from the donor animal. Following the connection of the cannulae to the perfusion device and the initiation of NMP, the graft was subjected to a thorough examination for any signs of bleeding. In this experimental setup, the microdialysis catheter (Model 63, M Dialysis AB, Stockholm, Sweden) was inserted through a sterile bore into the graft storage container of the NMP device and placed into the liver parenchyma using an introducer. The catheter was then fixed to the liver parenchyma by use of sutures. (Figure 1)

*Sample analysis*

The concentrations of piperacillin were determined by HPLC-UV using a Prominence LC20 modular HPLC system equipped with an SPD-M30A PDA detector (set to 225) and LabSolution software (Shimadzu, Duisburg, Germany). Piperacillin was determined using a Cortecs T3 2.7 µm 100x3 mm column (Waters, Eschborn, Germany) preceded by a guard column (Nucleoshell RP18 2.7µm 4×3 mm, Macherey-Nagel, Düren, Germany). The mobile phase consisted of 0.02 M sodium phosphate buffer/acetonitrile 80:20 (v/v), pH 7. At a flow rate of 0.4 mL/min and a column temperature of 40°C, piperacillin eluted after 4.7 minutes. Sample preparation was performed as previously described.[5] In brief, total drug concentrations in NMP perfusate were determined after protein precipitation with acetonitrile and removal of acetonitrile by extraction into dichloromethane. Free drug concentrations in NMP perfusate were determined after ultrafiltration using NMP perfusate (300 µL) buffered with 3 M potassium phosphate, pH 7.4, (10 µL) and Vivafree 500 30 kD Hydrosart centrifugal ultrafiltration devices (Vivaproducts Inc., Littleton, MA, USA) as previously described. Microdialysate was injected directly. Bile (20 µL) was diluted 1:50 with 25 mM sodium dihydrogen phosphate (980 µL) and then injected directly. Injection volumes were 1 µL for all matrices.

The linearity was shown from 0.1-300 mg/L in human serum as surrogate for NMP perfusate (R >0.998) and from 0.03-300 mg/L in saline as surrogate for microdialysate, ultrafiltrate and diluted bile (R >0.999). Based on in-process quality controls (QCs, spiked human serum from healthy volunteers), the intra- and inter-assay imprecision of the determination of total piperacillin in plasma was <7% (coefficient of variation, CV), the mean accuracy was 103%. The unbound fraction of piperacillin in these QCs (human serum) was 87 ± 2.9% corresponding to an inter-assay precision of 3.3% (CV).

*Measurement parameters*

Non-compartmental pharmacokinetic analysis was performed using Phoenix WinNonlin 8 (Certara, Princeton, NJ, USA). The elimination rate constant λ_z_ was determined by log-linear regression in the elimination phase and typically included the time interval from 3 to 8 hours for NMP perfusate and 3.5 and 8.5 hours for microdialysate. The linear-up log-down trapezoidal rule was used to calculate AUC_8h_. Extrapolation to infinity to obtain AUC_inf_ was based on the last predicted concentrations at 8 or 24 h.

The unbound fraction (*f*_u_) was calculated as *f*_u_ = C_free_/C_total_. Prism 9 (GraphPad Software, La Jolla, CA, USA) was used to calculate statistics. Given the relatively small sample size and the resulting uncertainty regarding the distribution of the data, the non-parametric Wilcoxon test was used to compare parameters between perfusate and interstitial fluid. Although non-parametric tests were applied, data are presented as mean ± standard deviation (SD) to facilitate comparison with previously published pharmacokinetic studies. A two-sided p-value < 0.05 was considered significant.

1. Wagner J, Mathis S, Spraider P, Abram J, Baldauf S, Pinggera D, et al. The effects of bolus compared to continuous administration of adrenaline on cerebral oxygenation during experimental cardiopulmonary resuscitation. Resusc Plus. 2024; 19:100738. DOI: <https://doi.org/10.1016/j.resplu.2024.100738>

2. Nasralla D, Coussios CC, Mergental H, Akhtar MZ, Butler AJ, Ceresa CDL, et al. A randomized trial of normothermic preservation in liver transplantation. Nature. 2018; 557:50-6. DOI: <https://doi.org/10.1038/s41586-018-0047-9>

3. Ravikumar R, Jassem W, Mergental H, Heaton N, Mirza D, Perera MT, et al. Liver Transplantation After Ex Vivo Normothermic Machine Preservation: A Phase 1 (First-in-Man) Clinical Trial. Am J Transplant. 2016; 16:1779-87. DOI: <https://doi.org/10.1111/ajt.13708>

4. Cardini B, Oberhuber R, Fodor M, Hautz T, Margreiter C, Resch T, et al. Clinical Implementation of Prolonged Liver Preservation and Monitoring Through Normothermic Machine Perfusion in Liver Transplantation. Transplantation. 2020; 104:1917-28. DOI: <https://doi.org/10.1097/TP.0000000000003296>

5. Kratzer A, Schießer S, Matzneller P, Wulkersdorfer B, Zeitlinger M, Schlossmann J, et al. Determination of total and free ceftolozane and tazobactam in human plasma and interstitial fluid by HPLC-UV. J Pharm Biomed Anal. 2019; 163:34-8. DOI: <https://doi.org/10.1016/j.jpba.2018.09.044>
